# Evaluation of anterior maxilla bone condition using CBCT for placing dental implant

**DOI:** 10.6026/9732063002001038

**Published:** 2024-09-30

**Authors:** Nureldeen AN Elhammali, Pushkar Gupta, Sohini Deb, Amit Chhaparwal, Rinkee Mohanty, Samarth Tiwari, Naveen Reddy R.

**Affiliations:** 1 Department of Oral and Maxillofacial Surgery, Faculty of Dentistry, Sirte University, Libya; 2 Department of Prosthodontics and Crown & Bridge, Hitkarini Dental College and Hospital, Jabalpur, MP, India; 3 Kalinga Institute of Dental Sciences, Bhubaneswar, Odisha, India; 4 Department of Conservative Dentistry and Endodontics, Geetanjali Dental College & Research Institute, Udaipur, Rajasthan, India; 5 Department of Periodontics, Institute Of Dental Sciences, SOA Deemed to be University, Bhubaneswar, Odisha, India; 6 Department of Periodontics, Index Institute of Dental Sciences, Malawanchal University, Indore, MP, India; 7 Department of Prosthodontics, College o-f Dentistry, Jazan University, Saudi Arabia

**Keywords:** Bone, maxilla, dental implant, undercut

## Abstract

Careful planning is essential for a successful outcome of dental implants. Determining the size of the implant and placement angle requires precise knowledge of the alveolar bone's height, width, shape, and density surrounding the intended implant location. Hence the goal of the current research was to use cone beam computed tomography (CBCT) to evaluate the anterior maxilla's bone state for dental implant insertion. The study included 30 patients, both male and female, who had CBCT scans of their anterior maxilla and needed dental implants in their maxillary anterior teeth. Measuring parameters included buccal undercut position and depth, as well as bone height and width. When comparing the canine region to the incisors, the mean bone height and width was higher. Buccal undercut, however, was more for the incisor region. The difference was statistically significant (P<0.05).

## Background:

These days, implant insertion is a frequent procedure used to restore lost teeth. It is inevitable for soft tissue and bone to resorb after tooth loss, which could seriously compromise the volume of remaining bone and compromise the placement of implants [1]. It has been demonstrated that alveolar bone resorption happens after tooth extraction regardless of delayed or immediately implants placement [2]. The results of dental implant reconstruction and the surgical preparation for the implantation process both benefit from anatomical pattern analysis of the bone. Implant therapy is most appropriate for patients whose cortical bone thickness around a cancellous bone is sufficient. Regardless of the dentate state, bone thickness in the coronal levels is lower and more prone to resorption than at the apical sections [1]. When planning a dental implant procedure, the anterior maxilla needs to be carefully taken into account [3]. Implant treatment planning used to be standardised using traditional radiography methods as cephalometric, panoramic, and intraoral radiographs. However, the precision of treatment planning using these strategies is compromised by superimposition and image distortion. The application of tomographic technology in the examination of possible implant sites is supported by advancements in sectional imaging techniques. Cone beam computed tomography (CBCT) has gaining importance in dentistry now, which opening up new possibilities for precise surgical guidance and thorough preoperative evaluation of implant sites. Accurate and high-resolution multiple planed reformatted images can be produced by CBCT [4]. Cone-beam computed tomography (CBCT) facilitates the determination of anatomical indices for implant placement as well as the morphological properties of the remnant alveolar bone [5]. When compared to computed tomography (CT), cone beam computed tomography (CBCT) produces osseous anatomy of the oro-maxillofacial area at a substantially lower effective radiation dosage [2]. Because of its quick scanning time, low dose, low cost, and superior resolution to CT scanning, CBCT is frequently used in dentistry for diagnostic and treatment planning [1]. Viewing a comprehensive three-dimensional picture of the area of interest is another advantage of CBCT [6].

Bone height loss following tooth extraction is frequently caused by dimensional changes, particularly in the alveolar plate of the face following implant placement. Furthermore, some research suggests that the width and shape of the alveolar bone, in addition to the height of accessible bone, influence the outcome of dental implants [6]. In order to create a treatment plan for implant repair, the clinician must assess the quality of the bone prior to surgery. Precise data on bone density is essential for determining appropriate implant locations, implant architecture, and surgical techniques. The quantity of bone in the edentulous region taken into account for implant osseointegration is known as available bone [2]. Sufficient thickness of the alveolar bone is known to protect the blood supply following extraction, nourish the socket created by the extraction, and stop avascular necrosis in the peri-implant bone. It is often advised to maintain the thickness of the 2-mm alveolar bone in order to reduce the possibility of biological and cosmetic issues. The diagnostic evaluation is crucial because persistent marginal bone resorption may have a deleterious effect on the cosmetic results of implant cases that are initiated right away. For the purpose of determining appropriate or optimal bone in three dimensions, lingual bone crest measurements are crucial [5]. It is well recognised that the coronal region of the alveolar bone has a more significant influence on the final outcomes, both functionally and aesthetically, of implant restorations [1]. Therefore, it is of interest to assess the bone condition of anterior maxilla for dental implant placement using Cone Beam Computed Tomography (CBCT).

## Materials and Methods:

The present study consisted of 30 patients of both genders requiring dental implant in maxillary anterior teeth. Ethical approval was obtained from the concerned authority. Written consent was obtained from all the participants. Following a comprehensive oral examination, the anterior maxilla was scanned using a CBCT system. Patients underwent CBCT scans on a single machine at the i-CAT Next Generation Scanner; all images were acquired using standard parameters (120kvp; 5ma; Exposure time 4sec; Voxel spacing 0.4mm). A sensor detects the transmitted x-rays, and software transfers the data to a computer where it is reconstructed into three-dimensional images. The acquired pictures were used to measure parameters such bone height, bone width, buccal undercut depth, and location. The ANOVA test was used for statistical analysis of the resulting data. A P value of less than 0.05 was deemed significant.

## Results:

The average bone height was 17.34 mm in the canine region, 17.12 mm in the lateral incisor region, and 22.12 mm in the central incisor region (Table 1). The average bone width was 8.87 mm in the canine region, 7.79 mm in the lateral incisor region, and 9.54 mm in the central incisor region. The canine area had the greatest bucco lingual width at the crest and the lowest thickness of the labial bony plate at the crest for the lateral incisors. The difference is statistically considerable (Table 1). The average buccal undercut placement was 5.54 mm at the canine, 3.31 mm at the lateral incisor, and 5.11 mm at the central incisor. The buccal undercut depth was 0.62 mm at the central incisor, 0.64 mm at the lateral incisor, and 0.61 mm at the canine area (Table 2). The difference is statistically considerable (P<0.05).

## Discussion:

Teeth replacement with dental implants is a common practice. It is crucial to have enough alveolar bone volume and mesiodistal dimensions in the implant site in order to achieve the best possible functional and cosmetic restoration following implant treatment [1]. Careful planning is essential for a successful outcome with implants. Details on the alveolar bone surrounding the suggested implant site, including its height, width, shape, and density is necessary before implant placement [4]. One of the predictive criteria in evaluating the alveolar volume available for implant placement after extraction is the alveolar dimension previous to tooth extraction [7]. The restoration of the maxillary anterior region with implant-supported prostheses has been characterised as a complex process due to the presence of multiple local risk factors and the high aesthetic standards and expectations of patients. Furthermore, compared to the posterior portion, the anterior maxilla's alveolar ridge is narrower and has thinner cortical plates [6]. The lack of bone needed to sustain dental implants, especially the buccal bone for immediate implants in anterior teeth, is one issue that implantologists typically deal with [8]. In comparison to incisors, the canine region has the largest bone height and width, according to the current study. Anterior maxilla was evaluated by Kalla *et al.* For dental implant insertion. They concluded that the maximal bone height and width were seen in the canine region. In the central incisor area, the maximum buccal undercut was seen [9]. The outcome is consistent with what we discovered. Dina and colleagues assessed the alveolar bone's size and shape in the canine, lateral, and maxillary areas. According to this study, the canine had the highest values of bone height, whereas the right central incisor had the lowest values. These conclusions relate to our findings. Dana *et al.* showed no correlation between the age of the subjects and the maxillary concavity depths [6]. In comparison to other maxillary anterior teeth, Zhang *et al.* found that the lateral incisor at the anterior maxilla has the weakest alveolar bone and frequently has a buccal undercut that is the closest to the alveolar ridge [4].

Affendi *et al.* Present a new categorisation for anterior mandibular teeth related to immediate implant placement (IIP), quantify alveolar bone morphology, and show the connection between tooth angulation and alveolar bone thickness [5]. The quality of maxillary bone in the local population surrounding the maxillary central incisors was found to be weak and impaired in comparison to other groups, according to Shah *et al.* assessment of the alveolar ridge and buccal undercut dimensions at the maxillary central incisors [10]. The labial bone thickness (LBT) was examined at various levels in respect to the six anterior maxillary teeth by alali *et al.* There is no relationship between age or gender and the LBT in the six maxillary anterior teeth, which is primarily thin (<1 mm) [11]. The canine region would have the lowest chance of experiencing a buccal plate perforation in the anterior maxilla, whereas the lateral incisor region would have the highest risk. The lateral incisor exhibits the highest frequency of buccal undercut and the weakest alveolar ridge [7]. According to Aljabr *et al.* the fact that the alveolar bone thickness in the labial anterior region is less than 2 mm indicates that accurate bone measurement is crucial for a predictable outcome during implant implantation [12]. The prognosis of immediate implant insertion in the anterior maxilla has been shown to depend critically on the measurement of labial bone thickness [13]. Following tooth damage, Hirani *et al.* evaluated the clinical results of placing implants right away in newly extracted anterior maxilla sites. They came to the conclusion that, implants placed right away into newly created extraction sites can provide a reliable course of treatment with excellent implant survival rates [14]. The maxilla's cancellous bone had the lowest density, according to Hassan *et al.*, but the mandible's alveolar bone breadth was greater in the maxilla [8]. It has been proposed that one of the most important indicators of hard tissue defects or soft tissue recessions could be the existence of an insufficient buccal plate prior to tooth extraction. When implants are placed, these inadequacies become extremely problematic, especially in the highly attractive anterior maxillary region. In intermediate to advanced cases, the use of CBCT should be required for the treatment plan since implants placed without it could cause the operator to miscalculate the condition of the bone [15]. For long-term success, evaluation of the morphology of the alveolar bone is required prior to implant implantation. An early assessment of bone quality can benefit from CBCT evaluation. Additional research is required to verify the results.

## Conclusion:

Data shows that the maximal bone height and width were seen in the canine region, whereas the central incisor area showed the greatest buccal undercut. A CBCT evaluation can direct implant placement and aid in the preliminary evaluation of bone quality.

## Figures and Tables

**Table 1:** Measurement of bone height and width

**Bone measurement (mm)**	**Central incisor region**	**Lateral incisor region**	**Canine region**	***P***
Bone height	17.34	17.12	24.12	0.1
Bone width	8.87	7.79	9.54	0.1
Bucco-lingual width at crest	5.1647	5.0485	6.3536	0
Thickness of labial bony plate at Crest	0.7574	0.6235	0.8463	0.1

**Table 2:** Buccal undercut location and depth

**Bone measurement (mm)**	**Central incisor region**	**Lateral incisor region**	**Canine region**	***P***
Buccal undercut location	5.54	3.31	5.11	0.01
Buccal undercut depth	0.62	0.64	0.61	0.02
